# 

**Published:** 1866-12

**Authors:** 


### INDEX TO VOLUME VII.

PAGE.

Abscess in Testicle. By M. M. Eaton, M.D., 548
Advertisements, Medical. By N. S. Davis, M.D., 1
Alcohol and its Relations to Man, 413
Amaurosis and deafness of Smokers and Drinkers. By MM. Sichel & Triquet, 164
Ammonia, Muriate of, in Iritis. By J. S. Hildreth, M.D., 106
Anæsthesia, New Method of Producing. By B. M. Richardson, M.D., 487
Anæsthetics, 40, 173, 360
Andrews, E., on Climate, &c., 737
Anscultation and Percussion, Manual of, 751
Arrow Wounds, Notes on. By E. Cowes, M.D., 350
Arteries, Minute function of. By J. S. Jewell, M.D., 449
Association, State and National, 113
" American Medical, 119, 186, 235, 247, 367, 402

Barth, M., and M. Henri Roger on Ascultation, 751
Blepheritis Ciliaris. By E. Williams, M.D., 166
Blood-letting, 68, 129, 198, 262, 321, 385, 513
Blood Disease. By E. B. Stevens, 20
Bowman, J. E., on Practical Chemistry, 752
Brainard, Prof. Daniel, 50
Bromide of Ammonium, its Uses, 717
Bromine and its Compounds, 710

Book Notices:—

Annandale, on Malformations, &c. 234
Ashton, on Diseases, &c. 44
Barker, on Laughing Gas, 627

PAGE.

Bartholomew, on Spermatorrhœa, 568
" on Bromide Potass, 111
Burral, on Cholera, 375, 499
Canniff, on Principles of Surgery, 317, 498
Chapman, on Diarrhœa and Cholera, 317, 500
Dixon, on Diseases of the Eye 627
Echeverria, on Reflex Paralysis, 499
Elderhest, on Blowpipe Analysis, &c., 686
Farre, on Materia Medica, &c. 628
Flint, on Exploration of Chest, 686
" on Practice and Principles of Medicine, 233
" on Physiology of Man, 235
Garrat, on Medical Electricity, 567
Hammond, on Wakefulness, 45
Mackall, on Physical Force, 235
" on Life in Nature, 235
" on Law of Muscular Action, 235
Nelson, on Cholera, 499
Physician's Visiting List, 628
Radcliffe, on Epilepsy, 47, 234
Rand, on Elements of Medical Chemistry, 686
Roberts, on Urinary and Renal Disease, 235
Sansom, on Action and Administration of Chloroform, 43
Semeleder, on Rhinoscopy and Laryngoscopy, 47
Syme, on Principles of Surgery, 45
Tanner, on Practice of Medicine, 43
West, on Diseases of Infancy and Childhood, 112
Winslow, on Diseases of Brain and Mind, 110
Williams, on Ophthalmic Science, 375, 499

PAGE.
Camphorated Oil in Erysipelas, -- 729
Cancer and Consumption, relations to Climate, -- 737
Cancer Curers, -- 752
Carcinoma, with Perforation, -- 722
Cases, Clinical. By N. S. Davis, M.D., -- 433
Cataract, Extraction of. By G. Hay, M.D. -- 35
Cerebro-Spinal Meningitis. By J. S. Jewell, M.D., -- 589, 658
Cerebro-Spinal Meningitis, -- 705
Chapman, on Cholera, -- 317
Cheeney, B. H., on Camphorated Oil, &c., -- 729
Chemistry, Practical, with Analysis, -- 752
Chicago, Sanitary Condition of. By N. S. Davis, M.D., -- 10, 631
Chicago Medical Society, -- 753
Cholera, 31, 193, 251, 292, 299, 317 397, 470. 506, 553, 577, 636
" Hospitals, -- 599
Cholera and Suppression of Urine, 746
Cities, Public Health of, -- 761
Clark, on Cholera, -- 292, 553
Clinical Instruction on Diseases of the Eye, -- 752
Clinton Medical Society, -- 486
Close of the Volume, -- 753
Coffee, Influence of, on Urea. By C. E. Squarey, M.R.C.S., -- 228
Coffee as a Deodorizer, -- 572
College, Chicago Medical, 118, 179, 500, 637
" Rush Medical, -- 118
" Medical Instruction, -- 178
Convention, Medical, -- 481
" of Representatives from Medical Colleges, -- 312
Cook County Hospital, Statistics, - 754
Copaiba deprived of its Smell, -- 121
Correspondence, Foreign. By C. A. Pope, M.D., -- 54
Croup, Treatment of, -- 39
Cryptogamous Origin of Intermittent Fevers, -- 698
Crystals, "Dumb Bell." By D. W. Flora, M.D., -- 257

Davis, N. S., on Cholera, -- 193, 746
Death of Prof. Brainard, -- 610
Deformaties of the Face. By E. Andrews, M.D., -- 465
Dexter, on Cholera, -- 577
Dexter, R., on Chronic Ulcers, -- 731

PAGE.
Diagnosis, Differential, -- 121
" of Curvature of Spine. By J. S. Sherman, M.D., -- 683
Dictionary, Biographical, -- 506
Digitalis, Action of, -- 252
Diphtheria, Discussion on, -- 402
" Treatment of, -- 443
Diseases of Children. Clinic By C. West, M.D., -- 150
Disease among the Freedpeople. By R. Reyburn, M.D., -- 494
Diseases, Prevalence of, &c. By N. S. Davis, M.D., -- 147, 475
Disinfection and Deodorization, -- 637
" Process of, -- 365
" Report on, -- 410

Entropion and Trichiasis. By J. S. Hildreth, M.D., -- 328
Epidemics, Influence of Electricity on, -- 410
Erysipelas, Camphorated Oil in, - 729
Esculapian Society, -- 743
Ether as a Local Application. By J. J. Black, M.D., -- 438
Eye and Ear Diseases in County Hospital, -- 246
Eye and Ear, Clinics on, -- 752
Explanation, -- 446

Farcy, Chronic, in Man, -- 61
Fees in Medical Colleges, -- 236
Fever, Causes of Remittent and Intermittent, -- 363
Fever, Typho Malarial. By W. H. Veatch, M.D., -- 666
Fistula, Vesico-Vaginal. By D. Mason, M.D., -- 400
Flora, on Cholera, -- 470
Fright, A Case of, -- 698

Galactozyme, -- 125
Given, A., on Case of Cancer, -- 722
Graduates, -- 246
Guaco as a Remedy in Cholera, -- 759

Hæmoptysis. By R. Payne Cotton, M.D., -- 357
Hatch, Ira, on Bromide of Ammonium, Therapeutic Uses, -- 717
Hemiphlagia, Facial, &c. By W. R. Saunders, M.D., -- 94
Hollister, J. H., on Bromine, &c.- 710
Hospital, Cook County, -- 49, 179
" Mercy, -- 245
" St. Luke's, -- 504

PAGE.
Hospital, Insane, of California, -- 250

Inhalation of Nebulized Fluids, -- 406
Instruments, Surgical, ---------- 571
Instruction, Clinical, in Chicago, - 629
Investigations of Mineral Waters. By M. Scoutetten, ------------ 60
Iritis, Muriate of Ammonia in, --- 106
Itch, Epidemic or Army. By S. C. Butler, M.D., ------------- 174

Journal, Chicago Medical, ------- 186

Lambelle, M. Jobert de, -------- 253
Lancet, London, ----------- 119, 247
Laryngoscopy, Laughing in. By H. A. Johnson, M.D., --------- 82
Letter and Prospectus, ---------- 378
Letter from D. Prince, M.D., ---- 83
Lime, Hydrate of, ------------- 444
Lithotomy, Four Operations on same Patient, --------------- 415

McWilliams, S. A., on Strychnine, 726
Medical Jurisprudence, Manual of, 751
Medical Societies, ------ 740, 743, 753
Membrana, Tympani, Artificial. By D. B. St. John Roosa, ----- 223
Meningitis, Cerebro-Spinal, ------ 116
Meningitis, Cerebro-Spinal, ------ 705
Meteorogical, Section of, &c., ---- 417
Millard, on Blood-letting in Fevers, 198
Morgan County Medical Society, - 420
Morgan County Medical Society, - 740
Mortality of Chicago, 123, 125, 246, 248, 381, 445, 505, 570, 571, 630, 692, ----------------------- 699
Mortality Statistics, --------- 759, 760
Mushrooms, Poisonous principle of, 698

Narcotism, -------------------- 569
Nasal Passages, Irrigation of. By H. A. Johnson, M.D., --------- 552
Neligan, J. Moore, on Diseases of Skin, ----------------------- 751
Nervous Affections. By C. E. Brown-Séquard, M.D., --------- 272
Neuralgia, Remedy for, --------- 253
News, European Medical, ------- 503
Nicotine, Baneful Effects of, Prevented, ---------------------- 588
Notice to Subscribers, ----------- 317

Opium. By S. B. Hawley, M.D., 76
Os Uteri, Dilitation of, by Incisions, ----------------------- 442

PAGE.
Ovariotomy. By E. B. Wolcott, M.D., --------------------- 76
Obituary of C. S. Tripler, M.D., U.S.A. ---------------------- 756
Obituary, Wm. B. Herrick, ----- 120
" I. P. Linn, ---------- 185
" R. D. Mussey, -------- 508
" Thomas Hodgkin, ----- 509
" W. T. Brand, --------- 571
" Daniel Brainard, ----- 687
" Record, ------------- 608

Payne, F. R., on Cerebro-Spinal Meningitis, -------------------- 705
Periodicals, Medical, ------------ 49
Periscope of Medical Science. By G. J. Ziegler, M.D., ----------- 88
Personal, --------------------- 446
Physician's Pocket Record, ------ 755
Physiology, Hygiene, &c., ------- 409
Poisoning by Strychnine, ------- 726
Poisons, Sale of, in Bombay, ---- 93
Poisonous Principle of Mushrooms, 698
Potassa, Permanganate of, ------- 411
Pregnancy, Tubal. By A. Fisher, M.D., ---------------------- 270
Prize Questions, ------------ 753, 757
Prize, Stevens' Triennial, -------- 507
Prizes of the Imperial Academy of Medicine, ------------------- 508
Psychology, Section on, &c., ----- 420
Ptelea Trifoliata, Medical Uses of. By O. F. Potter, M.D., -------- 298
Practical Medicine and Epidemics. By H. Noble, M.D., ---------- 528
Public Health of Cities, --------- 761

Quacks, Aurora Homeopathic, --- 376

Rectum, Diseases of the. By S. R. Lane, F.R.C.S., -------------- 27
Reports, -- 10, 147, 158, 250, 475, 504, 528, 631
Rinderpest, ---------------- 227, 380
Rock River Medical Society, ---- 484
Roger, M. Henri, on Ascultation, &c., ----------------------- 751

Sangamon Co. Medical Society, -- 691
Sarcocele Syphilitic, ----------- 444
Scurvy. By S. Hemmenway, M.D., ---------------------- 582
Sewage, Value of, -------------- 702
Skin, Diseases of, by J. M. Neligan, 751
Societies, --------- 420, 484, 486, 691
Spinal Curvature. By F. O. Earle, M.D., --------------------- 479

PAGE.
Statistics of the Profession in Paris, 366
Strychnine, Poisoning by, 726
Sulphites in Malarial Fevers, 503
Surgery, Section on, 413
Swinging as a Remedy, 762

Tapeworm. By J. V. Herriott, M.D., 587
Taylor, Alfred Swayne, on Medical Jurisprudence, 751
Thomas, Dr. T. G., 253
Tissues, Cellular Vitality of. By B. H. Cheeney, M.D., 538
Tooth. Diseased Effects of. By J. S. Hildreth, M.D., 201
Tooth, Diseased, connected with the Eye, 247
Trichinæ, 300, 315

PAGE.
Trichinæ, 745
Trimble, on Blood-letting, 129, 262, 321, 385, 573
Tripler, C. S., U.S.A., Death of, 756
Tumor, Encephaloid, 569

Ulcers, Chronic Treatment of, 751

Vaccine Matter from the Cow, 189
Vaccination and Syphilis, 125
" Compulsory, 411
Victim of the Risks connected with the Medical Profession, 125

White, Dr. J. P., 253

Young, on Blood-letting, 68
" on Cholera, 397

### BLISS & SHARP,

Wholesale & Ret[illegible]

### DRUGGISTS AND CHEMISTS,

**144 LAKE STREET, CHICAGO,**

Keep constantly on hand a large assortment of

### PURE DRUCS FINE CHEMICALS,

THAYER'S FLUID EXTRACTS & SUGAR-COATED PILLS,

**Keith's Preparations,**

Agents for the sale of

**Tiemann's Celeb ated Surgical Instruments,**

Trusses, Elastic Stockings, Bandages, &c.

**Particular Attention Paid to Physicians' O[illegible]ders.**

---

THE

### CHICAGO MEDICAL EXAMINER.

**N. S. DAVIS, M.D., EDITOR.**

A MONTHLY JOURNAL

DEVOTED TO THE

EDUCATIONAL, SCIENTIFIC, AND PRACTICAL INTERESTS

OF THE

**MEDICAL PROFESSION.**

The EXAMINER will be issued during the first week of each month, commencing with January, 1860. Each number will contain 64 pages of reading matter, the greater part of which will be filled with such contents as will directly aid the practitioner in the daily practical duties of his profession.

To secure this object fully, we shall give, in each number in addition to ordinary original articles, and selections on practical subjects, a faithful report of many of the more interesting cases presented at the Hospitals and College Cliniques. While aiming, however, to make the EXAMINER eminently practical, we shall not neglect either scientific, social, or educational interests, of the profession. It will not be the special organ of any one institution, society, or clique; but its columns will be open for well-written articles from any respectable member of the profession, on all topics legitimately within the domain of medical literature, science, and education.

Terms, $3.00 per annum, invariably in advance.

